# Improved multivariate modeling for soil organic matter content estimation using hyperspectral indexes and characteristic bands

**DOI:** 10.1371/journal.pone.0286825

**Published:** 2023-06-14

**Authors:** Ming-Song Zhao, Tao Wang, Yuanyuan Lu, Shihang Wang, Yunjin Wu

**Affiliations:** 1 School of Geomatics, Anhui University of Science and Technology, Huainan, Anhui, 232001, China; 2 Key Laboratory of Aviation-Aerospace-Ground Cooperative Monitoring and Early Warning of Coal Mining-induced Disasters of Anhui Higher Education Institutes, Huainan, Anhui, 232001, China; 3 Coal Industry Engineering Research Center of Collaborative Monitoring of Mining Area’s Environment and Disasters, Huainan, Anhui, 232001, China; 4 Nanjing Institute of Environmental Sciences, Ministry of Ecology and Environment of the People’s Republic China, Nanjing, 210042, China; 5 Key Laboratory of Soil Environmental Management and Pollution Control, Ministry of Environment Protection, Nanjing, 210042, China; ICAR Central Coastal Agricultural Research Institute, INDIA

## Abstract

Soil organic matter (SOM) is a key index of soil fertility. Calculating spectral index and screening characteristic band reduce redundancy information of hyperspectral data, and improve the accuracy of SOM prediction. This study aimed to compare the improvement of model accuracy by spectral index and characteristic band. This study collected 178 samples of topsoil (0–20 cm) in the central plain of Jiangsu, East China. Firstly, visible and near-infrared (VNIR, 350–2500 nm) reflectance spectra were measured using ASD FieldSpec 4 Std-Res spectral radiometer in the laboratory, and inverse-log reflectance (LR), continuum removal (CR), first-order derivative reflectance (FDR) were applied to transform the original reflectance (R). Secondly, optimal spectral indexes (including deviation of arch, difference index, ratio index, and normalized difference index) were calculated from each type of VNIR spectra. Characteristic bands were selected from each type of spectra by the competitive adaptive reweighted sampling (CARS) algorithm, respectively. Thirdly, SOM prediction models were established based on random forest (RF), support vector regression (SVR), deep neural networks (DNN) and partial least squares regression (PLSR) methods using optimal spectral indexes, denoted here as SI-based models. Meanwhile, SOM prediction models were established using characteristic wavelengths, denoted here as CARS-based models. Finally, this research compared and assessed accuracy of SI-based models and CARS-based models, and selected optimal model. Results showed: (1) The correlation between optimal spectral indexes and SOM was enhanced, with absolute value of correlation coefficient between 0.66 and 0.83. The SI-based models predicted SOM content accurately, with the coefficient of determination (*R*^*2*^) and root mean square error (*RMSE*) values ranging from 0.80 to 0.87, 2.40 g/kg to 2.88 g/kg in validation sets, and relative percent deviation (*RPD*) value between 2.14 and 2.52. (2) The accuracy of CARS-based models differed with models and spectral transformations. For all spectral transformations, PLSR and SVR combined with CARS displayed the best prediction (*R*^*2*^ and *RMSE* values ranged from 0.87 to 0.92, 1.91 g/kg to 2.56 g/kg in validation sets, and *RPD* value ranged from 2.41 to 3.23). For FDR and CR spectra, DNN and RF models achieved more accuracy (*R*^*2*^ and *RMSE* values ranged from 0.69 to 0.91, 1.90 g/kg to 3.57 g/kg in validation sets, and *RPD* value ranged from 1.73 to 3.25) than LR and R spectra (*R*^*2*^ and *RMSE* values from 0.20 to 0.35, 5.08 g/kg to 6.44 g/kg in validation sets, and *RPD* value ranged from 0.96 to 1.21). (3) Overall, the accuracy of SI-based models was slightly lower than that of CARS-based models. But spectral index had a good adaptability to the models, and each SI-based model displayed the similar accuracy. For different spectra, the accuracy of CARS-based model differed from modeling methods. (4) The optimal CARS-based model was model CARS-CR-SVR (*R*^*2*^ and *RMSE*: 0.92 and 1.91 g/kg in validation set, *RPD*: 3.23). The optimal SI-based model was model SI3-SVR (*R*^*2*^ and *RMSE*: 0.87 and 2.40 g/kg in validation set, *RPD*: 2.57) and model SI-SVR (*R*^*2*^ and *RMSE*: 0.84 and 2.63 g/kg in validation set, *RPD*: 2.35).

## Introduction

Visible and near-infrared reflectance (VNIR, 350–2500 nm) spectra of soil can reflect multiple soil properties, which can be effectively used for modeling prediction of soil properties [1–6]. Soil organic matter (SOM) is a key property determining soil functions and a major form of carbon stored in soil [4]. SOM content has a strong influence on soil reflectance characteristics. With the increase of SOM content, the spectral reflectance generally decreased in the VNIR spectra [4,7]. The chemical analysis of SOM content is time-consuming and costly, whereas VNIR spectral technology can quickly and accurately identify SOM content [3,8–12]. Various SOM prediction models were established in the different soil types and geographical regions based on many modeling methods [3,6,13–16], these models have achieved satisfactory accuracy.

In the processes of spectral prediction modeling, such as spectral preprocessing and transformation, spectral index extraction, characteristic band selection, and modeling methods, there are corresponding methods to improve the modeling accuracy. Spectral transformation, such as inverse-log reflectance (LR), continuum removal (CR), first-order derivative reflectance (FDR), and fractional order derivative *et al*., might improve the accuracy of SOM prediction model by enhancing the absorption or reflection features of soil spectra in some wavelengths [13,17–21]. For example, CR and FDR transformation had strong positive influence on the performance of most SOM prediction models [13,17]. FDR transformation showed better model performance than the second derivative transformation for SOM estimation in several modeling methods [18]. Continuous wavelet transform improved the accuracy and stability of the SOM predicted model significantly [22,23].

Spectral index was mainly calculated from two bands of continous hyperspetral data through algebraic operation, *i*.*e*. deviation of arch (DOA), difference index (DI), ratio index (RI), normalized difference index (NDI), and modified normalized difference index (MNDI) [14,24–26]. And then optimal spectral indexes were selected according to correlation with SOM and used for modeling SOM content. Hong *et al*. [25] reported that combination of two-dimensional spectral index (RI, DI and NDI) and extreme learning machine could rapidly and relatively accurately identify SOM level. Zhang *et al*. [26] reported that the combination of fractional-order derivative and MNDI could weaken the soil noise and improve the prediction accuracy. Zhao *et al*. [27] selected the optimal RI, DI and NDI calculated from continous VNIR spectra and establish the relatively accurate SOM inversion model. Compared with the predicted model using full spectral data, the modeling accuracy based on spectral index was acceptable, and the modeling variables were fewer.

Characteristic band screening aims to eliminate the uninformative variables and select characteristic bands from hyperspectral data using some algorithms and criterions. After band screening, the number of full spectral band is compressed significantly, which reduces the number of variables and the complexity of models during modeling process. Modeling precision based on selected characteristic bands usually higher than that based on the full spectra [10,28–30], or the dimensionality of the spectra can be significantly reduced while assuring modeling precision [31]. The competitive adaptive reweighted sampling (CARS) algorithm, uninformative variables elimination algorithm, successive projections algorithm, uniform-interval wavelength reduction, and partial least squares regression (PLSR) based variable importance projection were widely used methods [10,28,30,32,33]. CARS algorithm selects characteristics bands with high absolute regression coefficient values in the PLSR model [32]. Some studies reported that CARS could compressed the number of original spectral wavelengths to lower than 16% [30,34]. CARS algorithm was an effective way to reduce the number of inputs and improve the PLSR, random forest (RF) and support vector regression (SVR) modeling accuracy of SOM [28,34,35]. Bao *et al*. [30] improved the SOM prediction accuracy based on CARS and optimal soil grouping strategy.

Many modeling methods, such as PLSR [3,8,36], multivariate adaptive regression spline [11,37], machine learning [11,13,38,39], deep learning [40], were used for predicting SOM content. Through systematic comparison of modeling accuracy, these models can yield acceptable accuracy of SOM prediction in different soil types and geographical areas, of which PLSR model has relatively high accuracy and robust prediction, and is widely used [11,13,20,36]. Machine learning methods, such as SVR and RF models, are also widely used for spectral modeling for SOM. SVR and RF are capable to model complex, non-linear and linear relationships between variables [11]. Many studies found that SVR model had the robust and accurate prediction, and the RF model performed relatively weak in spectral modeling SOM [11,13,34,41]. However, other studies showed that RF model or RF model combined with CARS accurately predicted SOM [30,42].

As mentioned above, soil hyperspectral data includes a large number of continuous bands, whereas there is different correlation and redundancy information among the wavelengths. During modeling of soil properties, calculating spectral index and screening characteristic bands are two common means for reducing redundancy information of hyperspectral data. Both prediction model based on spectral index and characteristic band can accurately predict SOM content. However, there are few comparisons of the improvement of model accuracy by spectral index and characteristic band screening.

Against this background, soil samples in a typical farming area of East China were selected as study object. After different spectral transformations of VNIR spectral data of soil, optimal two-dimensional spectral index (RI, DI and NDI) and DOA were calculated and selected, and characteristic bands were selected by the CARS. Then SOM prediction models were established based on RF, SVR, deep neural networks (DNN) and PLSR models, using optimal spectral index and characteristic bands, respectively. The objectives of this study were to: (1) compare improvement of modeling accuracy by spectral index and characteristic band, (2) analyze the influence of the CARS algorithm on the accuracy of the RF, SVR, DNN, and PLSR models, (3) assess the modeling performance of RF, SVR, DNN and PLSR models, and establish optimal SOM prediction model in East China.

## Materials and methods

### Study area and soil sampling

**Fig 1:**
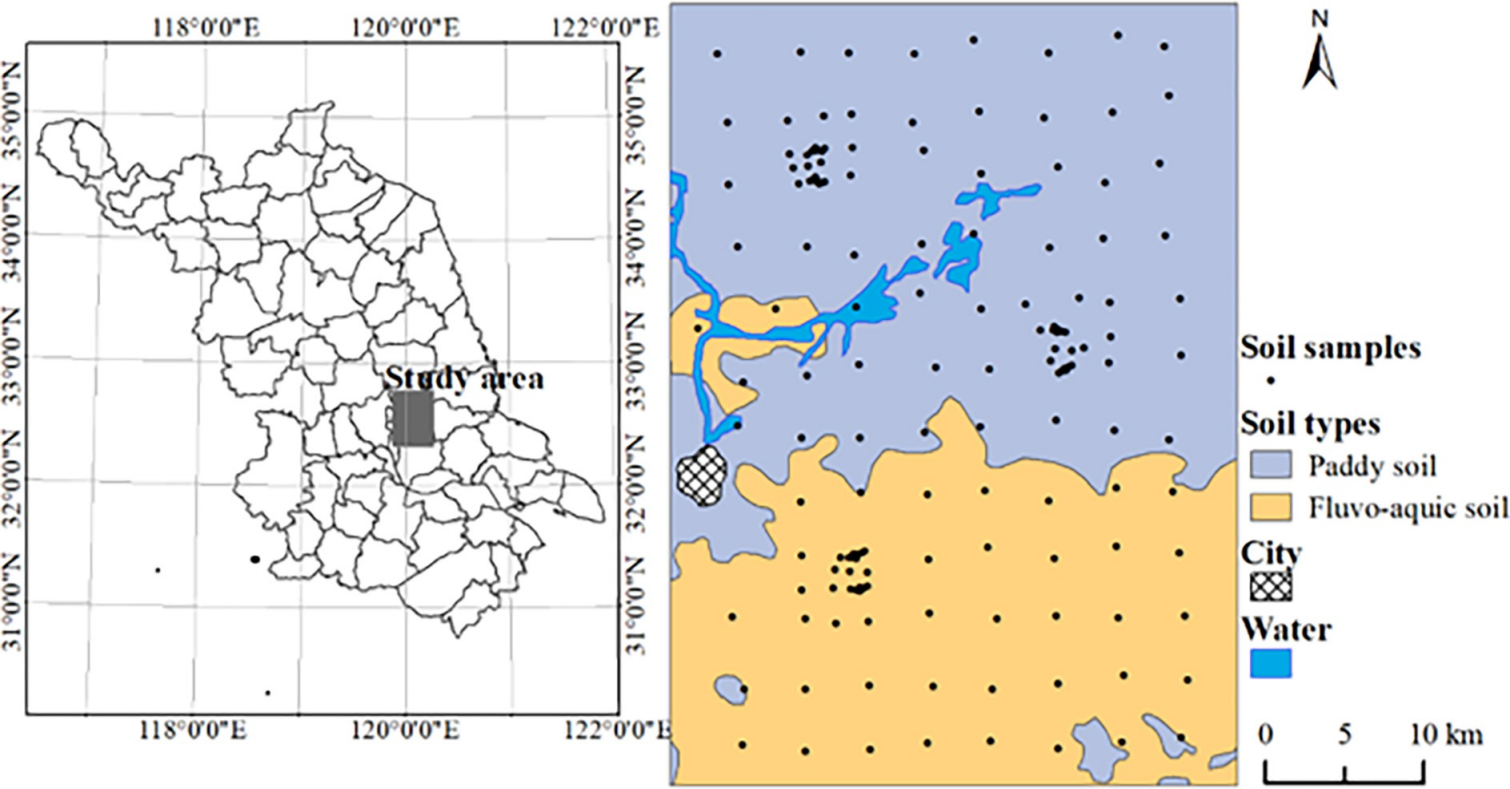
Location of study area and sampling sizes.

Study area is located in the central plains of Jiangsu Province (119°53′37″–120°14′4″ E, 32°20′17″–32°44′50″ N) of East China, covering an area of 1350 km^2^ (Fig 1). The annual average temperature and precipitation are 14.5°C and 990 mm, respectively. The elevation ranged from 5 to 10 m, with an increasing trend from south to north of study area. Parent materials mainly include lagoonal facies sediments. Paddy soil and paddy fields are the dominant soil types and land use type. The rice-rape rotation is the main crop rotation system.

A total of 178 soil samples were collected from the surface layer (0–20 cm) in November 2009 (Fig 1). In each field, 8–12 soil samples were collected within a radius of 10–20 m from the field center. The collected soil samples were mixed and 1 kg was retained using the quartation method. After soil samples were air-dryed and grinded in the lab, and then part of soil was sieved using a 0.2-mm soil sieve and used to measure SOM content. The SOM content was analyzed by the potassium dichromate method, the same as wet oxidation [43].

### Soil spectrum collection and preprocessing

After air-drying, grinding and sieving (< 2 mm), the diffuse reflectance spectra of the soil samples were measured using an ASD FieldSpec 4 portable spectral radiometer (Analytical Spectral Devices Inc., Boulder, USA). The wavelength range, and resampling interval are VNIR (350–2500 nm) and 1 nm, respectively. The entire operation was performed in the dark laboratory with controlled lighting condition with the light source of halogen lamp. The soil samples were placed in containers with a diameter of 10 cm and a depth of 1.5 cm, and the surface of sample was scraped flat. The sensor probe was located 15 cm above the surface of the soil sample vertically, with view angle of probe of 25°. The spectrometer was calibrated using a white panel with 99% reflectance before measuring. Each sample was rotated four direction, 10 scannings from each direction. Hence, 40 scanning spectral curves were collected for each sample and the mean was used as the spectra of the soil sample [7].

The Savitzky-Golay (SG) filter method with a moving window of 11 nm and a local polynomial order of 2 regression was used to smooth the reflectance spectra. LR, CR, and FDR were applied to transform the raw spectra (R) to enhance the relationship between the SOM and the spectra. Finally, each soil sample got 2141 bands for each type of spectral data in the VNIR (355–2495 nm) range. Spectral data processing was performed using “*prospectr*” package [44] in R software.

### Spectral index calculation

In this study, four selected spectral indexes were as follows: DOA [24,45], DI, RI, and NDI [14,25]. DOA was calculated using the reflectance of soil at 550, 600 and 650nm. SOM content was negatively correlated with DOA [24,27,45]. If the soil has rich SOM content, the reflectance spectral curve of soil shows a flat state in the range of 550–650 nm with low DOA value. According to Eqs (2)–(4), with the use of spectral bands in the 355–2495 nm range, DI, RI, and NDI were calculated. The contour map of absolute value of the correlation coefficient between SOM and calculated spectral indexes were analyzed to select optimal spectral indexes for estimating SOM. The spectral indexes were calculated as the follows:

$$DOA=R_{600}-(R_{550}+R_{650})/2$$ (1)

$$DI=R_{i}-R_{j}$$ (2)

$$RI=R_{i}/R_{j}$$ (3)

$$NDI=(R_{i}-R_{j})/(R_{i}+R_{j})$$ (4)

where *R*_500_, *R*_600_, and *R*_650_ are raw spectral reflectance at the wavelength of 500 nm, 600 nm, and 650 nm; *R*_*i*_ and *R*_*j*_ represent the reflectance values at wavelengths *i* and *j* in the range of 355–2495 nm, respectively. The spectral index was calculated and selected in MATLAB R2012a.

### Characteristic band screening algorithm

The CARS algorithm selects characteristics variables with high absolute values of regression coefficient in the PLSR model. It consists of three major steps, including Monte Carlo sampling, PLSR modeling, and acquisition of variable weights. This algorithm executes forced band selection by exponential damping function and makes competitive band selection using the adaptive reweighted sampling technique. The detailed process of CARS is shown in the reference [32]. CARS algorithm was executed in MATLAB R2012a.

### Modeling methods

**Fig 2:**
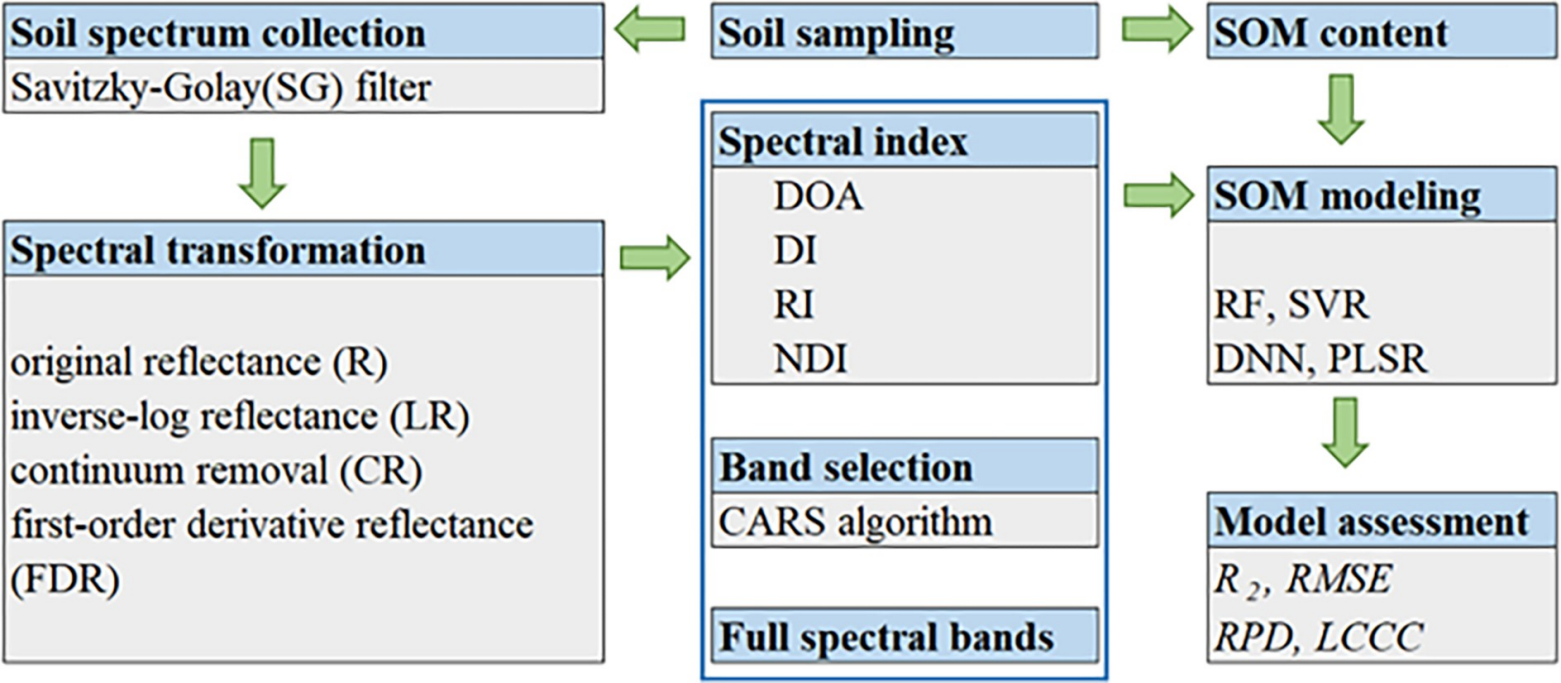
The flowchart of this research.

178 soil samples were divided into a calibration set (133 soil samples) and a validation set (45 soil samples) using the Kennard-Stone method [46]. After spectral transformation, spectral indexes and characteristic bands were selected. Firstly, spectral indexes were calculated according to Formulas (1)–(4). DOA was calculated using R spectrum, DI, RI and NDI were calculated using R, LR, CR and FDR spectra, respectively. Secondly, characteristic bands were selected from R, LR, CR, and FDR spectra by CARS algorithm, respectively. Thirdly, the SOM predicted models were established using spectral indexes and characteristic bands, respectively. RF, SVR, DNN and PLSR models were selected as modeling methods. Finally, model accuracy assessment and comparative analysis were conducted. Fig 2 was the flowchart for this research.

### Random forest (RF) and support vector regression (SVR)

In RF modeling, the two main parameters were the number of trees to grow in the forest (*n*_*tree*_*)* and the number of randomly selected predictor variables at each node (*m*_*try*_). In SVR modeling, the linear kernel function was used to build the model and the main parameter was the penalty coefficient (*C*). The parameters *m*_*try*_ and *n*_*tree*_ were set to 1–5 and 100–2000 for RF modeling, *C* range was set to 2^−4^–2^4^ for SVR modeling, and “*e1071*” package [47] of R software was used for parameter tuning using grid search and 10-fold cross-validation.

#### Deep neural networks (DNN)

DNN also known as multi-layer perceptron (MLP), are extensions of neural network [48–50]. The basic idea is to construct a multi-layer neural network model by modifying and increasing the number of hidden layers. Generally, the DNN model can be divided into three parts: input layer, hidden layer and output layer. The model used in this paper is a deep network model based on H2O learning platform [51]. The first layer of the whole DNN model is the input layer, namely the training sample or test sample, while the output layer is the solution of classification or regression problem, and the hidden layer is between the input layer and the output layer. For the DNN model, the ReLU function was selected as the activation function, parameters such as hidden layer, number of neurons, L1 regularization were adjusted successively to establish the optimal model, and the training times were selected as 1000. The hidden layer sizes were set as 400, 600 and 500; and the hidden layer dropout ratios were set as 0.9, 0.8 and 0.7. Due to the small number of samples we used the 10-fold cross-validation to train the model in this study. DNN modeling was performed using “*h2o*” package [52] in R software.

#### Partial least squares regression (PLSR)

PLSR was a popular method for quantitative analysis of hyper spectra. It is used to establish predictive models when many predictive variables were highly collinear. PLSR algorithm integrates the compression and regression steps and it selects successive orthogonal factors that maximize the covariance between predictor and dependent variables [11]. PLSR modeling was performed using “*pls*” package [53] of R software. The number of predictive variables to use in the PLSR models is selected by cross validation. Statistical analysis was performed using “*stats*” package [54] of R software.

### Model evaluation

The coefficient of determination (*R*^*2*^), root mean square error (*RMSE*), relative percent deviation (*RPD*), and Lin’s concordance correlation coefficient (*LCCC*) were used for model accuracy assessment. *RMSE* is smaller as *R*^*2*^ approaches 1, indicating better stability and higher prediction precision of the model. According to three levels of *RPD* classified by Chang *et al*. [55], when *RPD* is smaller than 1.4, the models have poor estimation capability; when *RPD* is between 1.4 and 2.0, the estimation precision of the models is improved to some extent; when *RPD* is grater than 2, the models achieve considerably high precisions. *LCCC* displays the distribution and aggregation degree of predicted and measured values near the 1:1 line, and the larger the value is, the better.

The calculation formulas of the evaluation indexes were as follows:

$$R^{2}=1‐{\sum_{i=1}^{n}\left(O_{i}‐P_{i}\right)}^{2}/{\sum_{i=1}^{n}\left(O_{i}‐\bar{O}\right)}^{2}$$ (5)

$$RMSE=\sqrt{\frac{1}{n}{\sum_{i=1}^{n}\left(O_{i}‐P_{i}\right)}^{2}}$$ (6)

$$RPD=s_{o}/RMSE$$ (7)

$$LCCC=2rs_{o}s_{p}/[s_{o}^{2}+s_{p}^{2}+{\left(\bar{O}‐\bar{P}\right)}^{2}]$$ (8)

where *O*_*i*_ and *P*_*i*_ are the observed and predicted value respectively. $\bar{O}$ and $\bar{P}$ is the mean value of observed and predicted value; *s*_*o*_ and *s*_*p*_ are the corresponding standard deviation; *r* is the correlation coefficient between the observed and predicted values. *n* is the number of observations.

## Results

### Characteristic of soil spectral curves

The SOM content ranged from 11.85 g/kg to 58.22 g/kg, with mean value of 28.54 ± 7.80 g/kg (Table 1). The coefficient of variation of SOM were 27.31%, belonging to moderate variation.

**Table 1:** Statistical characteristics of soil organic matter content.

Sample set	*n*	Range (g/kg)	Mean (g/kg)	Standard deviation	Skewness	Kurtosis	Coefficient of variation (%)
All samples	178	11.85–58.22	28.54	7.80	0.60	0.50	27.31
Calibration set	133	11.85–58.22	28.50	8.28	0.69	0.43	29.40
Validation set	45	14.42–40.61	28.67	6.24	-0.13	-0.54	21.77

**Fig 3:**
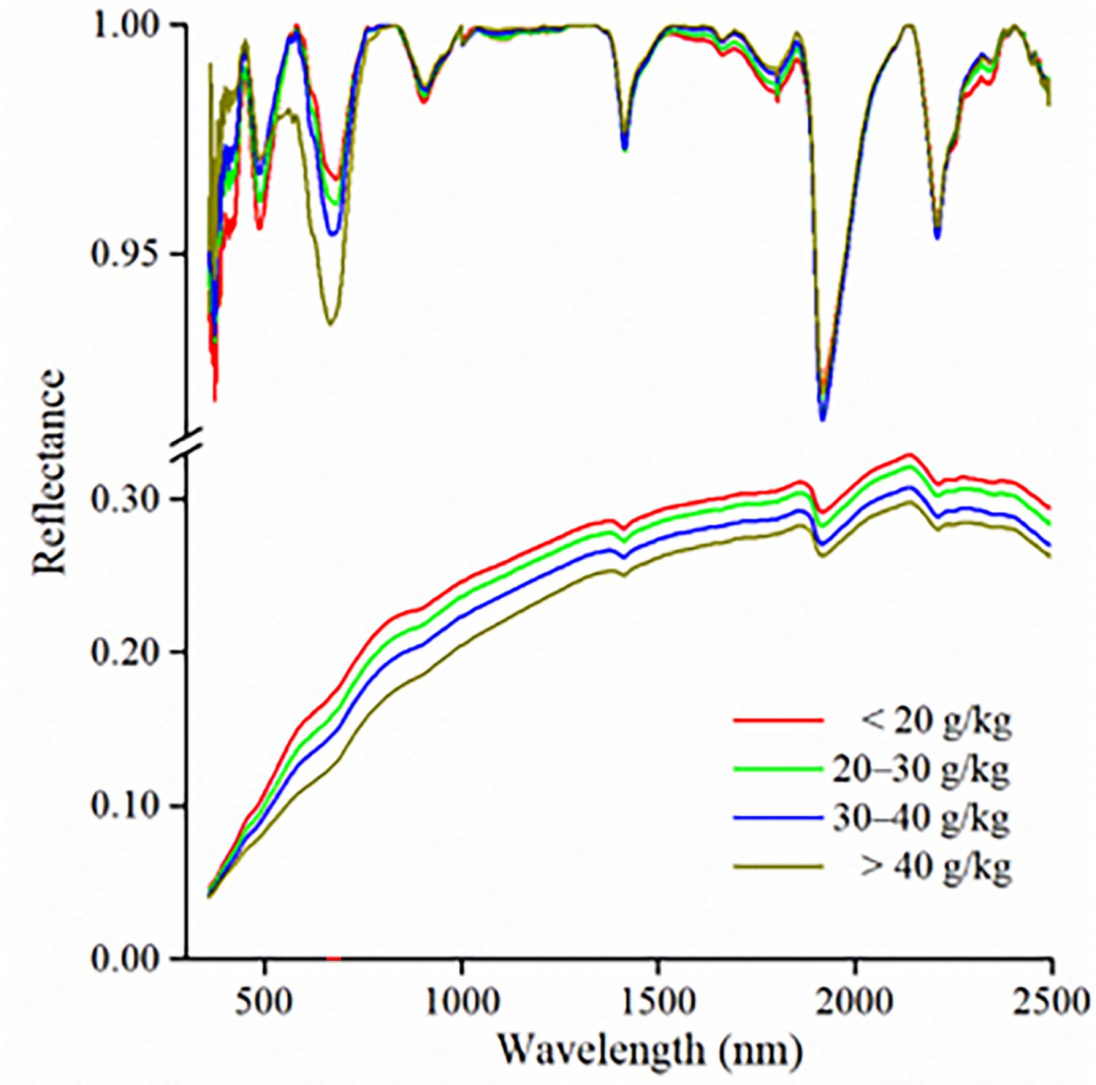
Soil mean spectral curves and continuum removal of different SOM content.

The SOM content was divided into four levels: < 20 g/kg, 20–30 g/kg, 30–40 g/kg and > 40 g/kg [39]. The mean spectral reflectance curves corresponding to four SOM content levels were calculated (Fig 3). With increasing SOM content, the spectral reflectance of the soil decreased over the full spectral range. With increasing wavelength, the reflectance in the visible spectrum increased quickly. In the NIR wavelength, the reflectance of soils was relatively high; however, it showed stable growth rate. The absorption characteristics were not apparent in the original spectral curves; however, after CR transformed, they were visibly strengthened and the depth of the absorption valley increased (Fig 3). Except for the more prominent absorption valleys near 1400 nm, 1900 nm, and 2200 nm, the relevant evident characteristics were also detected between 560 and 660 nm. The absorption characteristics near 650 nm were generally strengthened with an increase in SOM content. At the wavelength of 480 nm and 1650–1850 nm, and the depth of absorption valley became shallower with the increase of SOM content. After CR transformation, the absorption characteristics of soil spectral curves were more prominent, and the differences of spectral curves of different SOM contents were enhanced.

In NIR wavelengths, the molecular vibrations of NH-, CH-, and CO- were influenced by frequency doubling and combined frequency [56]. The absorption valley near 1400 nm was mainly caused by the soil surface adsorbed water and constitution water in the clay mineral O-H lattices. The absorption valley near 2200 nm was mainly attributed to the absorption wavelength of the Al-OH clay minerals [7,11].

**Fig 4:**
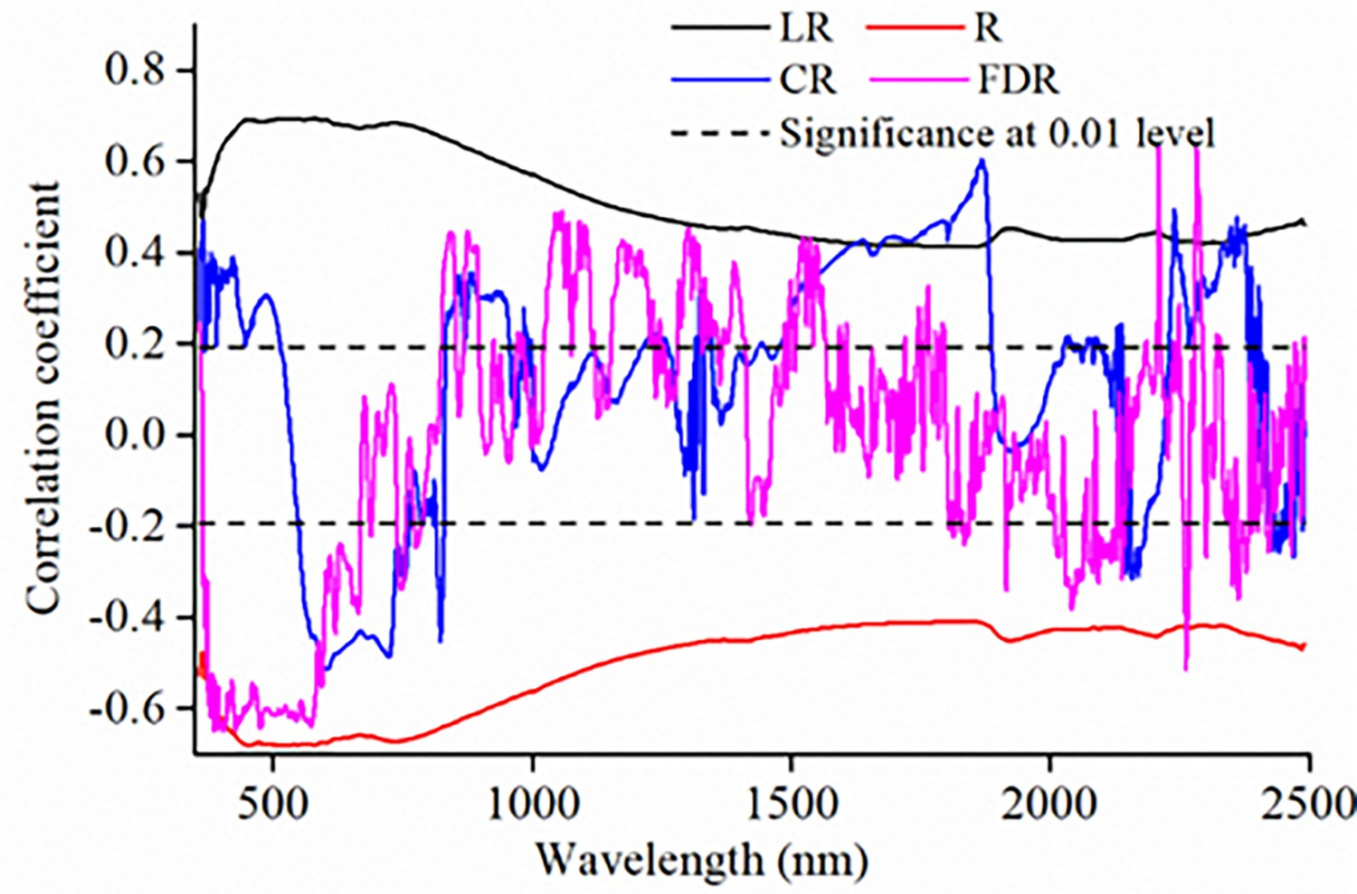
Correlation coefficient distribution between soil spectral data and SOM content.

SOM content showed significantly negative correlations with the R spectra in the full spectra range; however, a converse response pattern was observed for LR spectra (Fig 4). The correlations in the range of 400–900 nm were the strongest and the absolute values of the correlation coefficients were higher than 0.6 for R and LR spectra (Fig 4). The SOM and CR, FDR spectra presented significant positive or negative correlations at 400–750 nm, 1500–1700 nm, and 2200–2400 nm wavelengths, and the absolute values of correlation coefficient in the full spectral range were lower than those of the R and LR spectra (Fig 4). The strongest correlation between SOM content and R, LR, CR and FDR spectra were observed at 520 nm, 581 nm, 1869 nm and 428 nm, respectively, with the correlation coefficients of -0.68, 0.69, 0.60 and -0.65.

### Correlation between SOM content and spectral index

**Fig 5:**
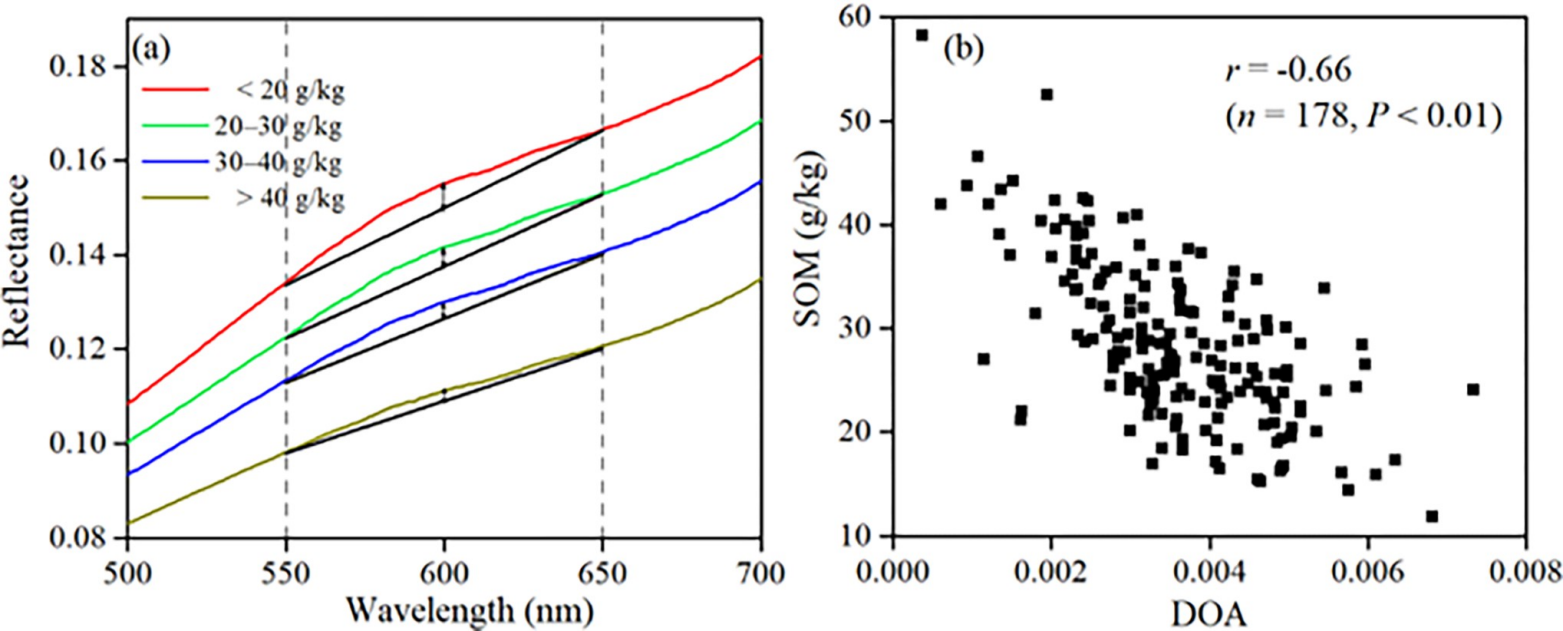
Relationship between SOM and deviation of arch related wavelengths.

The relationship between SOM and DOA of R spectra at 600 nm wavelength was shown in Fig 5. With the increase of SOM content, the original spectral curves gradually tended to be flat in the range of 550–650 nm, and the DOA gradually became smaller (Fig 5A). SOM content was significantly negatively correlated with the DOA value, with correlation coefficient of -0.66 (*p* < 0.01) (Fig 5B). The same findings were reported by Xu and Dai [45], Zheng *et al*. [24]. Zheng *et al*. [24] established four SOM predicted regression models based DOA in Coastal Soil, with the *R*^*2*^ values ranging from 0.38 to 0.51 and *RMSE* values ranging from 4.12 to 4.62 g/kg (*n* = 71). Gao *et al*. [57] compared SOM predicted models based on DOA of spectra at 600 nm and 800 nm respectively, and the predicted model with DOA at 600 nm displayed better accuracy, with the *R*^2^ value of 0.84 and *RMSE* of 4.57 g/kg (*n* = 53).

**Fig 6:**
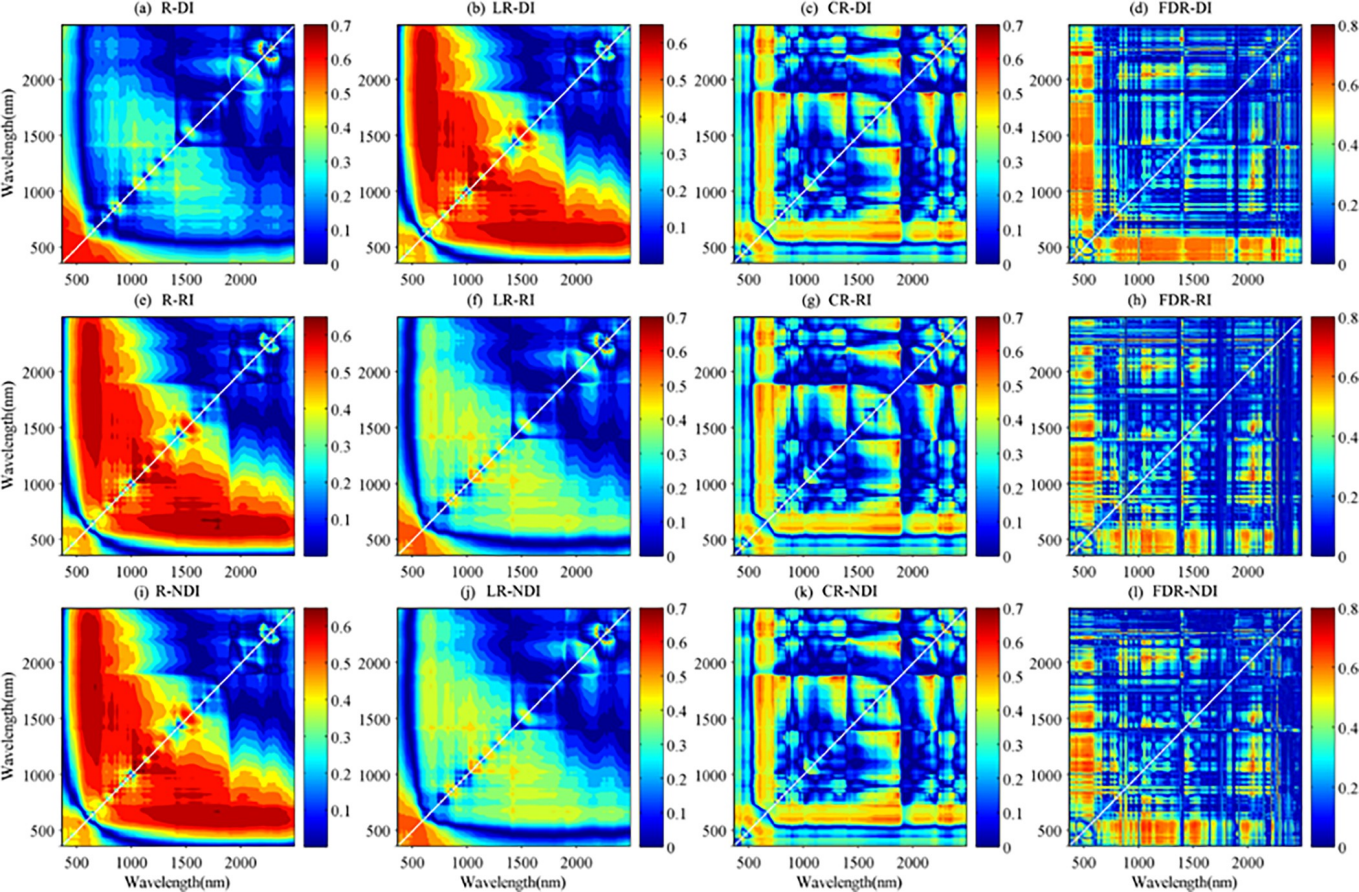
A contour map of the correlations between SOM content and spectral index (*n* = 178).

The correlations between SOM and RI, DI, and NDI for different spectral transformations were analyzed (Fig 6). The contour map showed the absolute value of the correlation coefficient between SOM and the spectral indexes. The x- and y-axes represent the bands of 355–2495 nm. Results showed that the good correlation of DI, RI, and NDI comprised of two bands to SOM mainly focused within 2200–2230 nm and 1390–1410 nm (Fig 6).

For example, in R, the good correlation between SOM and DI were observed in the range of 2200–2230 nm, with the absolute value of correlation coefficient greater than 0.7. The highest correlation coefficient between SOM and DI, RI, NDI derived from R were 0.73, 0.68, and 0.68, respectively, which was located at 2215 nm on x-axis and 2202 nm on y-axis (Table 2). Similarly, the optimal spectral indexes for LR, CR, and FDR spectra were extracted according to highest correlation with SOM (Table 2). The absolute value of correlation coefficient between the SOM and reflectance of bands composing optimal spectral indexes were between 0.10 and 0.44 (Table 3). After calculating DI, RI and NDI, the correlation with SOM was significantly improved, with the absolute value of the correlation coefficient exceeding 0.68 (Table 2). This result indicated that the correlation between spectral feature and SOM was enhanced after calculating the spectral index, and the accuracy of modeling was improved to different extent [25]. These optimal spectral indexes were used for modeling SOM content based on RF, SVM, DNN, and PLSR methods.

**Table 2:** The optimal spectral indexes selected by correlation for each spectral transformation.

Spectral index	Correlation coefficient	Spectral index	Correlation coefficient	Spectral Index	Correlation coefficient	Spectral index	Correlation coefficient
**DI**_**R(2215, 2202)**_	**0.73**	**DI**_**LR(2215, 2202)**_	**0.68**	**DI**_**CR(2223, 2182)**_	**0.71**	**DI**_**FDR(1408, 1396)**_	**0.80**
DI_R(2219, 2198)_	0.73	DI_LR(2214, 2203)_	0.67	DI_CR(2223, 2183)_	0.70	DI_FDR(1407, 1397)_	0.79
DI_R(2217, 2199)_	0.72	DI_LR(2215, 2203)_	0.66	DI_CR(2171, 1877)_	-0.70	DI_FDR(1408, 1395)_	0.78
**RI**_**R(2202, 2215)**_	**0.68**	**RI**_**LR(2215, 2202)**_	**0.72**	**RI**_**CR(2223, 2182)**_	**0.71**	**RI**_**FDR(1391, 2213)**_	**0.83**
RI_R(2215, 2202)_	0.68	RI_LR(2202, 2215)_	0.72	RI_CR(2223, 2183)_	0.70	RI_FDR(1393, 1403)_	0.82
RI_R(2203, 2214)_	0.67	RI_LR(2219, 2198)_	0.71	RI_CR(2171, 1877)_	-0.69	RI_FDR(2211, 1393)_	0.82
**NDI**_**R(2215, 2202)**_	**0.68**	**NDI**_**LR(2215, 2202)**_	**0.72**	**NDI**_**CR(2223, 2182)**_	**0.71**	**NDI**_**FDR(1403, 1393)**_	**0.82**
NDI_R(2214, 2203)_	0.67	NDI_LR(2219, 2198)_	0.71	NDI_CR(2223, 2183)_	0.70	NDI_FDR(1404, 1393)_	0.81
NDI_R(2215, 2203)_	0.66	NDI_LR(2217, 2199)_	0.71	NDI_CR(2171, 1877)_	-0.70	NDI_FDR(1404, 1392)_	0.81

Note: All correlation coefficients were significant at 0.01 level.

**Table 3:** The correlation between SOM and bands composing of optimal spectral indexes.

Wavelength (nm)	Correlation coefficient	Wavelength (nm)	Correlation Coefficient	Wavelength (nm)	Correlation coefficient
R_2198_	-0.44	LR_2203_	0.44	FDR_1392_	0.38
R_2199_	-0.44	LR_2214_	0.44	FDR_1393_	0.37
R_2202_	-0.44	LR_2215_	0.44	FDR_1395_	0.35
R_2203_	-0.44	LR_2217_	0.44	FDR_1396_	0.34
R_2214_	-0.44	LR_2219_	0.44	FDR_1397_	0.33
R_2215_	-0.44	CR_1877_	0.54	FDR_1403_	0.28
R_2217_	-0.44	CR_2171_	-0.31	FDR_1404_	0.26
R_2219_	-0.43	CR_2182_	-0.23	FDR_1407_	0.23
LR_2198_	0.44	CR_2183_	-0.22	FDR_1408_	0.22
LR_2199_	0.44	CR_2223_	0.10	FDR_2211_	0.39
LR_2202_	0.44	FDR_1391_	0.38	FDR_2213_	0.22

Note: All correlation coefficients were significant at 0.01 level.

## Results of characteristic band screening

**Fig 7:**
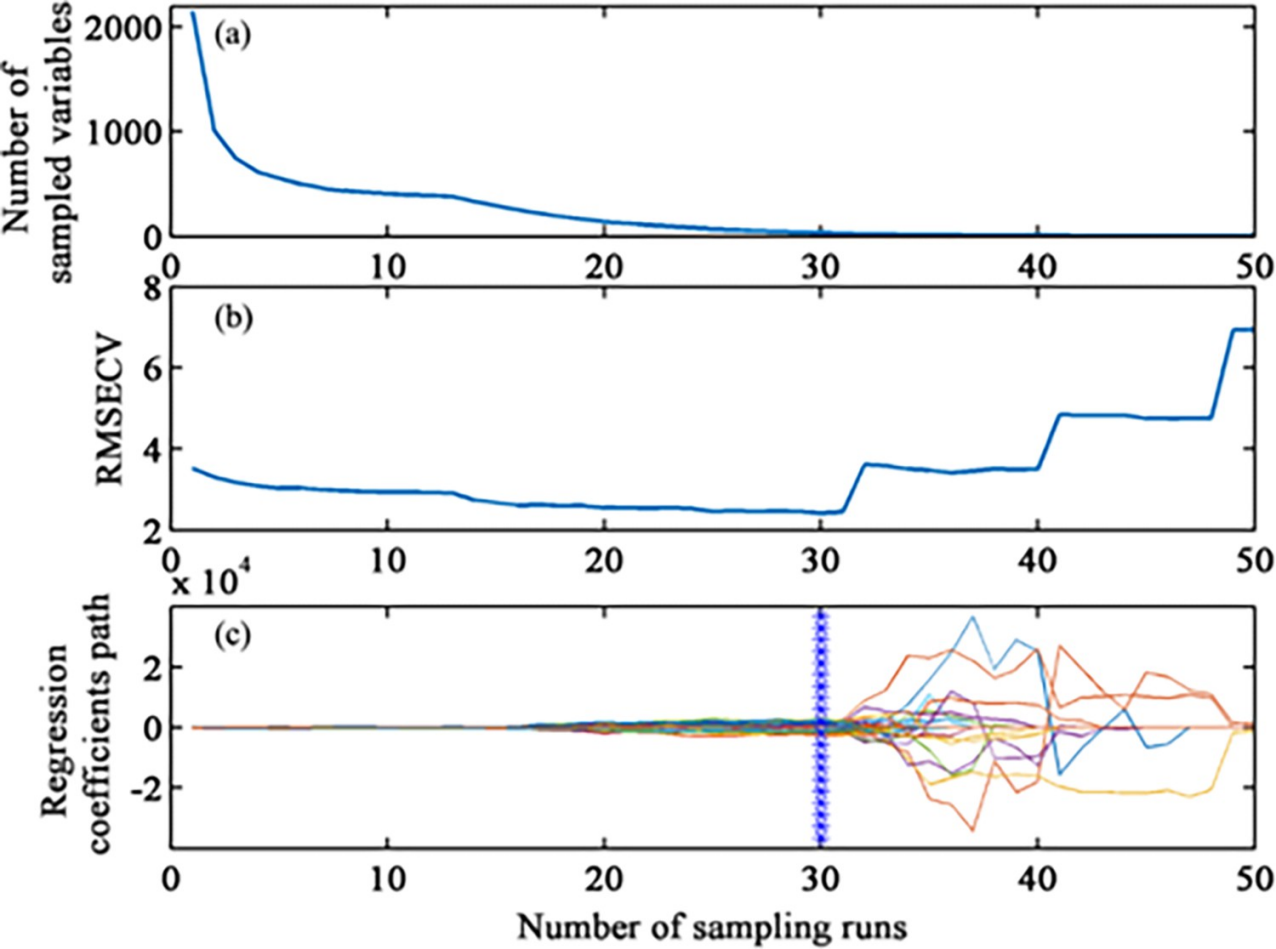
Key variables selected by CARS of raw spectra.

**Fig 8:**
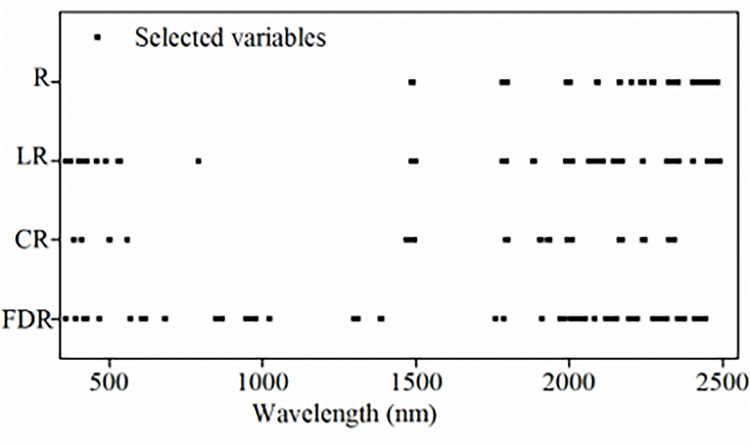
Plot of screened bands based on CARS for different spectral transformations.

Fig 7 showed the results of characteristic band screening for R spectra. The number of screened bands decreased continuously until reaching zero during the screening process, whereas the Monte Carlo sampling times increased continuously (Fig 7A). According to the trend of *RMSE* of cross-validation (RMSECV) (Fig 7B), the modeling accuracy increased, whereas the RMSECV decreased when the operation time increased from 1 to 30, caused by the remove of the wavelengths lowly correlated with SOM. At the 30th sampling time, RMSECV reached a minimum; therefore, the selected variable subset was the optimal. A total of 35 bands selected by the CARS algorithm were mainly distributed within 1990–2490 nm (Fig 8).

For LR, CR and FDR spectra, CARS algorithm screened 125, 46, and 81 bands from all 2141 bands, respectively. The selected bands were mainly distributed in the ranges of 400–900 nm and 1990–2490 nm, which was consistent with the research conclusions of Yu *et al*. [58], Tang *et al*. [35], and Bao *et al*. [30]. The CARS algorithm compressed the number of bands to lower than 6% of the full bands, and would reduce the complexity of SOM spectral modeling.

### Modeling of SOM content using spectral index

The SOM predicted models were established using the optimal spectral index (denoted here as SI-based model) (Table 4). Here are two optimal spectral index datasets, SI dataset included 13 spectral indexes with the highest correlation with SOM for each type of spectra, SI3 dataset included 37 spectral indexes with the three highest correlation with SOM for each type of spectra. These SI-based models (i.e., SI-RF, SI-SVR, SI-DNN, and SI-PLSR model) displayed the comparable accuracy. In calibration sets, the *R*^*2*^ value ranged from 0.80 to 0.85, *RMSE* value ranged from 3.21 to 3.69 g/kg. In validation sets, the *R*^*2*^ value ranged from 0.83 to 0.84, *RMSE* value ranged from 2.57 to 2.77 g/kg. The *RPD* values were both greater than 2, indicated that predicted models achieved considerably high precisions.

**Table 4:** SOM modeling results using optimal spectral index datasets.

Model ^a^	Calibration set		Validation set		*RPD*	*LCCC*
	$R_{c}^{2}$	*RMSE*_*c*_(g/kg)	$R_{p}^{2}$	*RMSE*_*p*_(g/kg)		
SI-RF	0.80	3.69	0.84	2.57	2.40	0.91
SI-SVR	0.85	3.24	0.84	2.63	2.35	0.91
SI-DNN	0.85	3.21	0.83	2.63	2.35	0.91
SI-PLSR	0.82	3.47	0.83	2.77	2.23	0.91
SI3-RF	0.80	3.67	0.86	2.45	2.52	0.92
SI3-SVR	0.89	2.75	0.87	2.40	2.57	0.93
SI3-DNN	0.85	3.19	0.83	2.61	2.36	0.91
SI3-PLSR	0.81	3.62	0.80	2.88	2.14	0.89

^a^ SI stands for spectral index dataset with the highest correlation with SOM for each type of spectra. SI3 stands for spectral index dataset with the three highest correlation with SOM for each type of spectra. Model SI-RF stands for RF modeling used SI dataset.

**Fig 9:**
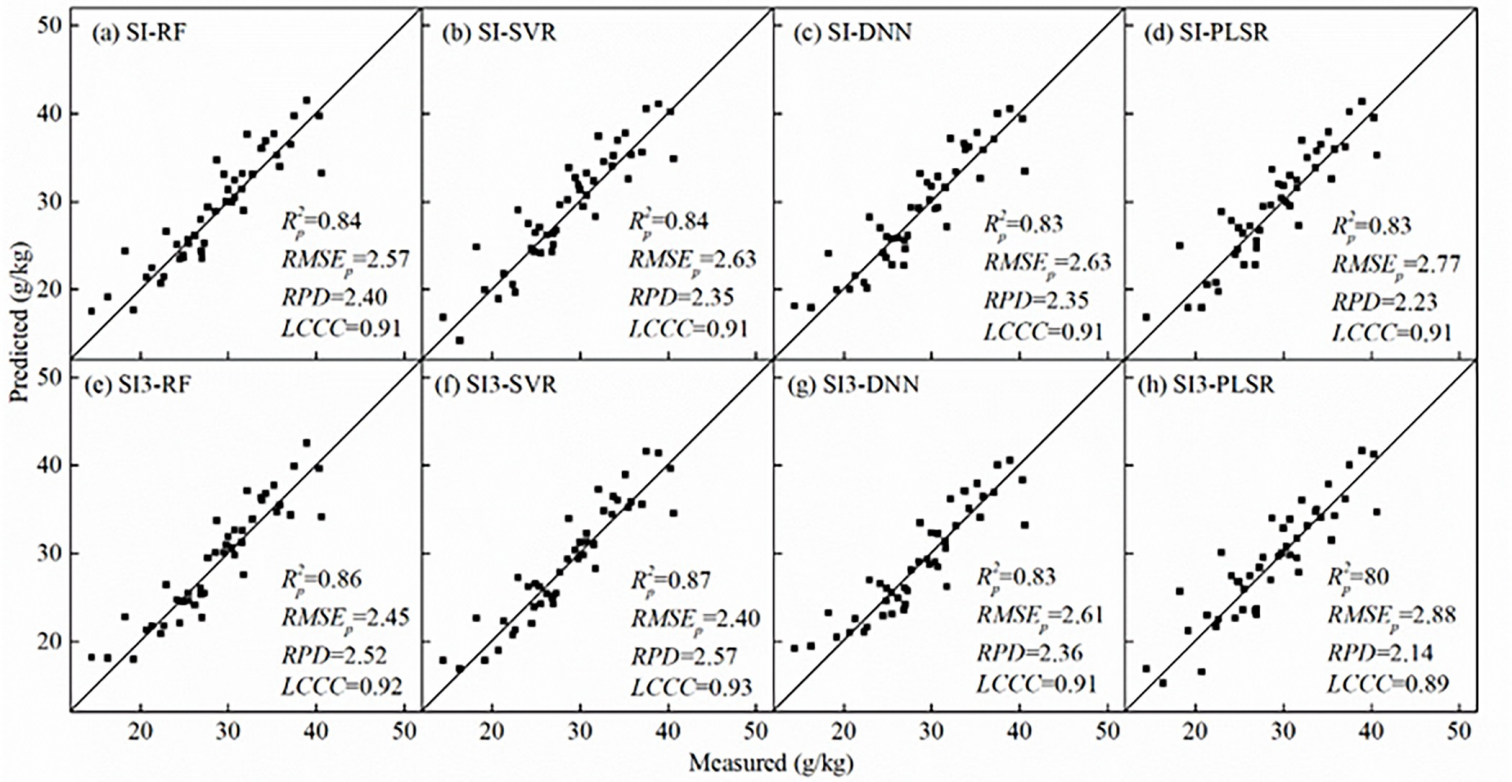
Scatter plots of measured and predicted SOM (*n* = 45).

The scatter plots of predicted and measured SOM were shown in Fig 9. High predictive precisions indicated that the predicted values are close to the measured values, near the 1:1 line. The *LCCC* values of four SI-based models were both 0.91, indicated that majority of scatter points were close to the 1:1 line. SI-RF model was slightly better than other models (with the highest *RPD* and *R*^*2*^ value, and the lowest *RMSE* value), whereas SI-PLSR model yielded the lower accuracy. Although more spectral index was added to models, these SI3-based models (i.e., SI3-RF, SI3-SVR, SI3-DNN, and SI3-PLSR model) did not significantly improved. The *R*^*2*^ and *RPD* value (except SI3-DNN and SI3-PLSR model) were only slightly larger than SI-based models, and the *RMSE* value were slightly lower than SI-based models. The distribution pattern in validation scatter plots were also very similar between SI-based models and SI3-based models (Fig 9).

Table 5 was the SOM modeling results using full spectral bands, of which SVR and PLSR models displayed better accuracy, with *R*^*2*^ values ranging from 0.72 to 0.87 in validation set, *RPD* value ranging from 1.87 to 2.79. SI-based models displayed the similar precision (Table 4), but the number of modeling variables were fewer, which were a total of 13 or 37 spectral indexes.

**Table 5:** SOM modeling results using full spectral bands.

Model ^a^	Calibration set		Validation set		*RPD*	*LCCC*
	$R_{c}^{2}$	*RMSE*_*c*_(g/kg)	$R_{p}^{2}$	*RMSE*_*p*_(g/kg)		
R-RF	0.52	5.71	0.25	5.36	1.15	0.58
R-SVR	0.94	2.03	0.86	2.34	2.64	0.93
R-DNN	0.68	4.70	0.25	5.33	1.16	0.61
R-PLSR	0.87	2.99	0.80	2.76	2.24	0.91
LR-RF	0.51	5.76	0.17	5.62	1.10	0.55
LR-SVR	0.96	1.78	0.87	2.21	2.79	0.94
LR-DNN	0.67	4.70	0.27	5.27	1.17	0.62
LR-PLSR	0.90	2.61	0.85	2.37	2.60	0.93
CR-RF	0.7	4.86	0.65	3.66	1.69	0.78
CR-SVR	0.99	0.76	0.72	3.27	1.89	0.87
CR-DNN	0.95	1.86	0.76	3.04	2.03	0.89
CR-PLSR	0.86	3.11	0.83	2.54	2.43	0.92
FDR-RF	0.69	4.82	0.52	4.28	1.44	0.72
FDR-SVR	0.99	0.85	0.82	2.65	2.33	0.92
FDR-DNN	0.95	1.87	0.82	2.65	2.33	0.91
FDR-PLSR	0.85	3.15	0.78	2.89	2.14	0.90

^a^ R, LR, CR, and FDR stand for different spectral data. Model R-RF stands for RF model using full bands of R spectra.

### Modeling of SOM content using characteristic bands

**Fig 10:**
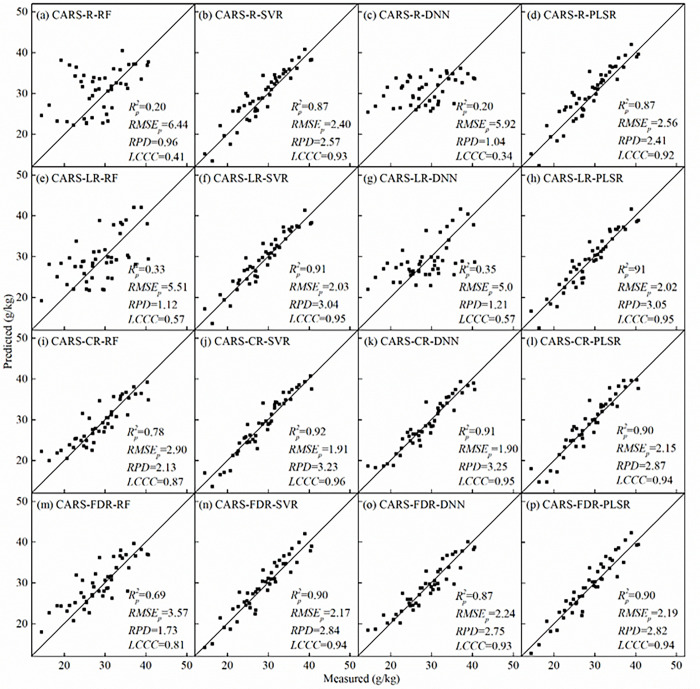
Scatter plots of measured and predicted SOM of CARS-based models (*n* = 45).

The SOM predicted models were established using the characteristic bands selected by CARS (denoted here as CARS-based model) (Table 6). The scatter plots of independent validation were shown in Fig 10. Compared with SOM predicted models using full spectral bands (Table 5), CARS algorithm was an effective way to reduce the number of inputs and improve the SVR and DNN modeling accuracy.

**Table 6:** SOM modeling results using characteristic bands.

Model ^a^	Calibration set		Validation set		*RPD*	*LCCC*
	$R_{c}^{2}$	*RMSE*_*c*_(g/kg)	$R_{p}^{2}$	*RMSE*_*p*_(g/kg)		
CARS-R-RF	0.15	7.91	0.20	6.44	0.96	0.41
CARS-R-SVR	0.93	2.17	0.87	2.40	2.57	0.93
CARS-R-DNN	0.24	7.20	0.20	5.92	1.04	0.34
CARS-R-PLSR	0.91	2.42	0.87	2.56	2.41	0.92
CARS-LR-RF	0.50	5.84	0.33	5.51	1.12	0.57
CARS-LR-SVR	0.95	1.91	0.91	2.03	3.04	0.95
CARS-LR-DNN	0.55	5.50	0.35	5.08	1.21	0.57
CARS-LR-PLSR	0.93	2.20	0.91	2.02	3.05	0.95
CARS-CR-RF	0.71	4.74	0.78	2.90	2.13	0.87
CARS-CR-SVR	0.92	2.39	0.92	1.91	3.23	0.96
CARS-CR-DNN	0.89	2.67	0.91	1.90	3.25	0.95
CARS-CR-PLSR	0.90	2.63	0.90	2.15	2.87	0.94
CARS-FDR-RF	0.76	4.34	0.69	3.57	1.73	0.81
CARS-FDR-SVR	0.96	1.66	0.90	2.17	2.84	0.94
CARS-FDR-DNN	0.94	2.09	0.87	2.24	2.75	0.93
CARS-FDR-PLSR	0.92	2.29	0.90	2.19	2.82	0.94

^a^ R, LR, CR, and FDR stand for different spectral data. Model CARS-R-RF stands for RF model using selected bands of R spectra.

For different spectral transformations, the PLSR models and SVR models with characteristic bands both obtained the best accuracy, of which SVR models were slightly better. The *R*_*c*_^*2*^ and *R*_*p*_^*2*^ values of SVR models (model CARS-LR-SVR, CARS-CR-SVR and CARS-FDR-SVR) were both greater than 0.9, the *RMSE*_*c*_ and *RMSE*_*p*_ values were lower than 2.40 g/kg. The *R*_*c*_^*2*^ values of PLSR models ranged from 0.90 to 0.93, *RMSE*_*c*_ values ranged from 2.20 to 2.63 g/kg; the *R*_*p*_^*2*^ values ranged from 0.87 to 0.91, *RMSE*_*p*_ values ranged from 2.02 to 2.56 g/kg (Table 6). The *RPD* values of SVR and PLSR models ranged between 2.41 and 3.23, the predicted and measured values were concentrated around the 1:1 line (Fig 10B, 10D, 10F, 10H, 10J, 10L, 10N and 10P), with the *LCCC* values ranging between 0.92 and 0.96, indicating considerably high accuracy and superior predictive capability of the SVR and PLSR models.

After FDR and CR spectral transformations, the DNN and RF models using characteristic bands achieved more accuracy than LR and R spectra. For example, the *R*_*p*_^*2*^ values of DNN and RF models (model CARS-FDR-RF, CARS-FDR-DNN, CARS-CR-RF and CARS-CR-DNN) ranged from 0.69 to 0.91, the *RMSE*_*p*_ values ranged from 1.90 and 2.24 g/kg (Table 6). The *LCCC* values ranging from 0.87 to 0.95 and the *RPD* values higher than 2.0 (Fig 10I, 10K, 10M and 10O), indicating good predictive ability of models. Generally, model CARS-FDR-DNN and CARS-CR-DNN were slightly more accurate than corresponding RF models. For LR and R spectral data, model CARS-LR-RF, CARS-LR-DNN, CARS-R-RF and CARS-R-DNN represented the worst predictive capability, with the *R*_*p*_^*2*^ values lower than 0.35, the *RPD* values lower than 1.4, and the *RMSE*_*p*_ values ranging from between 5.08 and 6.44 g/kg. The predicted and measured values were dispersedly distributed around the 1:1 line regardless of whether the lower or higher SOM content, the *LCCC* values ranged from 0.34 to 0.57 (Fig 10A, 10C, 10E and 10G).

From the above results, some useful conclusions can be drawn: (1) for the same spectral transformation, RF models (for example, model CARS-R-RF) had lower accuracy, while the SVR and PLSR models (for example, model CARS-R-SVR and CARS-R-PLSR) have higher accuracy; (2) for the same modeling methods, SOM predicted models using R (model CARS-R-SVR) had lower accuracy, models using FDR and CR (model CARS-FDR-SVR and CARS-CR-SVR) had better accuracy. Overall, this study showed that CR and FDR transformation could improved modeling accuracy, and CARS algorithm could improve the SVR and PLSR modeling accuracy, that was consistent with most research results [13,17,18,34].

## Discussion

### Improvement of modeling accuracy by spectral index

Extracting spectral indexes aims to recalculate new feature indexes of the original spectral data [24,25], and then building predicted models used optimal spectral indexes with stronger correlate to target variable. For example, commonly used spectral indexes were calculated from two bands via a mathematical method (some researches called two-dimension correlation coefficient), such as DI, RI and NDI, could enhance the relationship between SOM and spectral features [25]. Other optimal spectral indexes were also calculated from one band or three bands (some researches called one-dimension or three-dimension correlation coefficient) [19,25,26], such as MNDI.

Some previous studies established SOM prediction models using different spectral index, yielding acceptable prediction accuracy. For instance, Hong *et al*. [25] predicted SOM content accurately based on extreme learning machine model using optimal DI, RI, and NDI, with the *R*^*2*^ value larger than 0.8, the *RMSE* value lower than 5.0 g/kg. Zhang *et al*. [26] compared the sensitivity and estimation accuracy of SOM based on a single-dimensional index, two-dimensional index (NDI), and three-dimensional index (MNDI), respectively. MNDI exhibited the best model performance (with *R*^*2*^ value of 0.85, and *RMSE* of 4.02 g/kg in validation dataset), follewed by NDI model (with *R*^*2*^ value of 0.78, and *RMSE* of 4.80 g/kg in validation dataset). Zhao *et al*. [27] predicted SOM content based on linear regression model with DOA, DI, RI, and NDI, with the *R*^*2*^ value between 0.67 and 0.73, the *RPD* value between 1.79 and 1.94.

In this study, the SOM content were predicted accurately using SI-based models, with explanation 83% of the variation in SOM, and *RPD* value greater than 2.0. These results indicated that the correlation between spectral feature and SOM was enhanced after calculating the spectral index, and the accuracy of modeling was improved to different extent [25].

### Improvement of modeling accuracy by characteristic band

CARS algorithm could compress the number of full spectral band significantly [10,28,29,34]. Combination of CARS algorithm, the prediction models were remarkably improved [10,28–30]. For instance, Bao *et al*. [30] improved the SOM prediction accuracy based on CARS and optimal soil grouping strategy (*R*^*2*^ value between 0.76 and 0.89, *RPD* value between 2.02 and 2.97). Zhao *et al*. [34] estimated SOM content accurately based on PLSR and SVR models combining CARS (Paddy soil: *R*^*2*^ larger than 0.9, *RPD* larger than 2.0). In this study, the CARS algorithm reduced the number of bands considerably. The prediction accuracy of CARS-based models differed between modeling methods and spectral transformations. For all spectral transformations, PLSR and SVR models both obtained the best accuracy (*R*^*2*^ larger than 0.9, *RPD* larger than 2.0). For FDR and CR spectra, the DNN and RF models achieved more accuracy than LR and R spectra. In spectral inversion model of SOM, PLSR and SVR had the robust prediction and produced the smallest *RMSE* values, RF performed weakly [11,13,15,41].

CARS algorithm did not remarkably improve RF model accuracy [34], which was consistent with this research. This may be related to the principle of the CARS algorithm, which selects variables with relatively high absolute value of regression coefficient in the PLSR model [32]. However, there are some conflicting studies. Knox *et al*. [42] showed that RF model produced an *R*^*2*^ from 0.63 to 0.88, when using different spectral preprocessing only in the VNIR range. Bao *et al*. [30] showed that RF combined with CARS accurately predict SOM content, with the *R*^*2*^ values ranging from 0.65 to 0.89. The difference various in soil types and SOM content levels, might be another reason that RF models performed differently. In that study of Bao *et al*. [30], SOM content ranged from 4.25 to 80.32 g/kg, with mean of 39.5 ± 13.21 g/kg. In this study, RF models (model CARS-R-RF, CARS-LR-RF and CARS-FDR-RF) performed worse, with the *R*^*2*^ values ranging from 0.20 to 0.69, *RPD* values ranging from 0.96 to 1.73, SOM content was mean of 28.54 ± 7.80g/kg.

### Comparison of modeling accuracy by spectral index and characteristic band

Overall, the accuracy of SI-based models (*R*^*2*^ value between 0.83 and 0.87, *RPD* value between 2.14 and 2.57, *LCCC* value between 0.89 and 0.93) was slightly lower than that of CARS-based models (PLSR and SVR combining CARS and CR-DNN model: *R*^*2*^ value between 0.87 and 0.92, *RPD* value between 2.41 and 3.23, *LCCC* value between 0.92 and 0.96) (Tables 4 and 6). But the spectral index had a good adaptability to the models, and the accuracy of each SI-based model was similar, with *R*^*2*^, *RPD* and *LCCC* values almost equal. Characteristic band modeling has some selectivity to models, for different spectral transformation, the accuracy of each CARS-based model differed from modeling method to different degrees.

Compared with models using full spectral bands (Table 5), SI-based models displayed the similar precision (*R*^*2*^ and *RMSE* values were not significantly different) (Table 4). But the number of modeling variables were fewer, only 13 and 37 spectral indexes were selected for modeling. CARS-based models outperformed models using full spectral bands (Tables 5 and 6). A useful conclusion from this study is that both spectral index and characteristic band can reduce the number of modeling variables, improve modeling accuracy, or ensure that the modeling accuracy is not reduced.

## Conclusions

The correlation between spectral feature and SOM was enhanced after calculating the spectral index. For all spectral transformation, optimal spectral indexes were selected according to highest correlated with SOM. The SOM content were predicted accurately using optimal spectral index models, with explanation more than 83% of the variation in SOM, and *RPD* value greater than 2.0.

The CARS algorithm had a high compression ratio, and selected 35–125 characteristic bands from all wavelengths. The prediction accuracy of models combining CARS algorithm differed between modeling methods and different spectral transformations. For all spectral transformations, the PLSR and SVR combined with CARS algorithm displayed the best prediction (*R*^*2*^ and *RPD* values greater than 0.87 and 2.41). For FDR and CR spectra, the DNN and RF models achieved more accuracy than LR and R spectra.

Overall, the accuracy of SI-based models was slightly lower than that of CARS-based models. But the spectral index had a good adaptability to the models, and the accuracy of each SI-based model was similar. Characteristic band has some selectivity to models, the accuracy of each CARS-based model differed from models to different degrees. Compared with models using full spectral bands, both spectral index and characteristic band can improve modeling accuracy.

## Supporting information

S1 Data set (RAR)
